# Apolipoprotein A1 is associated with osteocalcin and bone mineral density rather than high-density lipoprotein cholesterol in Chinese postmenopausal women with type 2 diabetes mellitus

**DOI:** 10.3389/fmed.2023.1182866

**Published:** 2023-06-15

**Authors:** Wei Wang, Zhe Yuan Chen, Fen Yan Lv, Mei Tu, Xiu Li Guo

**Affiliations:** Longyan First Affiliated Hospital of Fujian Medical University, Longyan, Fujian, China

**Keywords:** osteoporosis, high-density lipoprotein, apolipoprotein A1, bone mineral density, bone turnover markers

## Abstract

**Objective:**

Disturbances in high-density lipoprotein cholesterol (HDL-c) metabolic pathways can affect bone metabolism, which may rely on the particle function of apolipoprotein rather than HDL-c levels. This study aimed to evaluate the correlation of serum HDL-c and apolipoprotein A1 (APOA1) with bone metabolism in Chinese postmenopausal women with type 2 diabetes mellitus (T2DM).

**Method:**

A total of 1,053 participants with complete data were enrolled and separated into three groups based on the HDL-c and APOA1 tertiles. The trained reviewer collected demographic and anthropometric information. Bone turnover markers (BTMs) were determined by standard methods. Bone mineral density (BMD) was measured by dual-energy x-ray absorptiometry.

**Results:**

Overall, the prevalence of osteoporosis was 29.7%. Groups with higher APOA1 have a remarkably more elevated level of osteocalcin (OC), L1-L4 BMD, and *T*-score across the APOA1 tertiles. APOA1 presented a positive correlation with OC (*r* = 0.194, *p* < 0.001), L1-L4 BMD (*r* = 0.165, *p* < 0.001), and *T*-score (*r* = 0.153, *p* < 0.001) rather than HDL-c. Meanwhile, APOA1 remained independently associated with OC (*β* = 0.126, *p* < 0.001), L1-L4 BMD (*β* = 0.181, *p* < 0.001), and *T*-score (*β* = 0.180, *p* < 0.001) after adjustment for confounding factors. APOA1 is also shown to be independently correlated with osteoporosis after adjustment for confounding factors, and the OR (95%CI) was 0.851 (0.784–0.924). In contrast, there was no significant association between HDL-c and osteoporosis. Furthermore, APOA1 seemed to have the largest areas under the curve (AUC) for osteoporosis. The AUC (95% CI) of APOA1 identifying osteoporosis was 0.615 (0.577–0.652). The optimal cut-off value of APOA1 was 0.89 g/L (sensitivity: 56.5%, specificity: 67.9%).

**Conclusion:**

APOA1 is independently associated with OC, L1-L4 BMD, and osteoporosis rather than HDL-c in Chinese postmenopausal women with T2DM.

## Introduction

Bone is a specialized connective tissue with several essential functions that can mechanically support the soft tissues, protect the internal organs and maintain calcium homeostasis. The maintenance of bone mass depends on the balance between bone formation and resorption. This balance ensures that bone can adapt to changes in mechanical loads and minor injuries. The activity of osteoblasts and osteoclasts plays a significant role in this balance. Notably, metabolic dysfunction can easily break bone metabolism homeostasis (1). Type 2 diabetes mellitus (T2DM) is a cluster of metabolic dysfunctions characterized by chronic hyperglycemia. The epidemiological survey showed that T2DM increases the risk of bone fragility fractures, and osteoporosis-related fractures have become major health concerns in T2DM (2–4). T2DM can break the bone metabolism homeostasis and accelerate the development of osteoporosis through some pathways, mainly including elevated advanced glycation end products (5), obesity (6), dyslipidemia (7), increased insulin resistance (8) and chronic inflammatory micro-environment (9) caused by T2DM. Among these mechanisms proposed to explain the association between T2DM and bone homeostasis imbalance, dyslipidemia with dysfunctional high-density lipoprotein cholesterol (HDL-c) involves the development of bone homeostasis imbalance (10).

In addition to the well-recognized anti-atherogenic effects, emerging evidence highlighted that HDL-c and its major protein component of apolipoprotein A1 (APOA1) also play more functional roles in other biological processes, including systemic inflammation, nitric oxide production, oxidative stress, and regulation of bone metabolism homeostasis (11). HDL-c is reported to be closely related to bone physiology and pathology. It was well-recognized that disturbances in lipid metabolic pathways can affect osteoblasts differentiation, leading to bone mass loss (12, 13). Nevertheless, the exact association between HDL-c level and bone mineral density (BMD) generated from epidemiological studies on humans remained uncertain and controversial. Genetic background, age, eating habits, hormonal and metabolic status, and HDL-c functionality relying on the particle function of apolipoprotein rather than HDL-c levels may be responsible for this inconsistency. Likewise, our previous study (14) enrolled 619 postmenopausal women with T2DM revealed that monocyte to APOA1 ratios had a higher area under the curve (AUC) value in identifying osteoporosis than the monocyte to HDL-c ratios, which indicate us that serum APOA1 may be more associated with osteoporosis rather than serum HDL-c. In addition, current epidemiological data on the associations between HDL-c, APOA1 level, and BMD in Chinese postmenopausal with T2DM are lacking. Hence, based on our previous study, we enrolled more participants to further evaluate the associations between HDL-c, APOA1, and BMD, bone turnover markers (BTMs) in Chinese postmenopausal women with T2DM.

## Study design and methods

### Study design and participants

**Figure 1:**
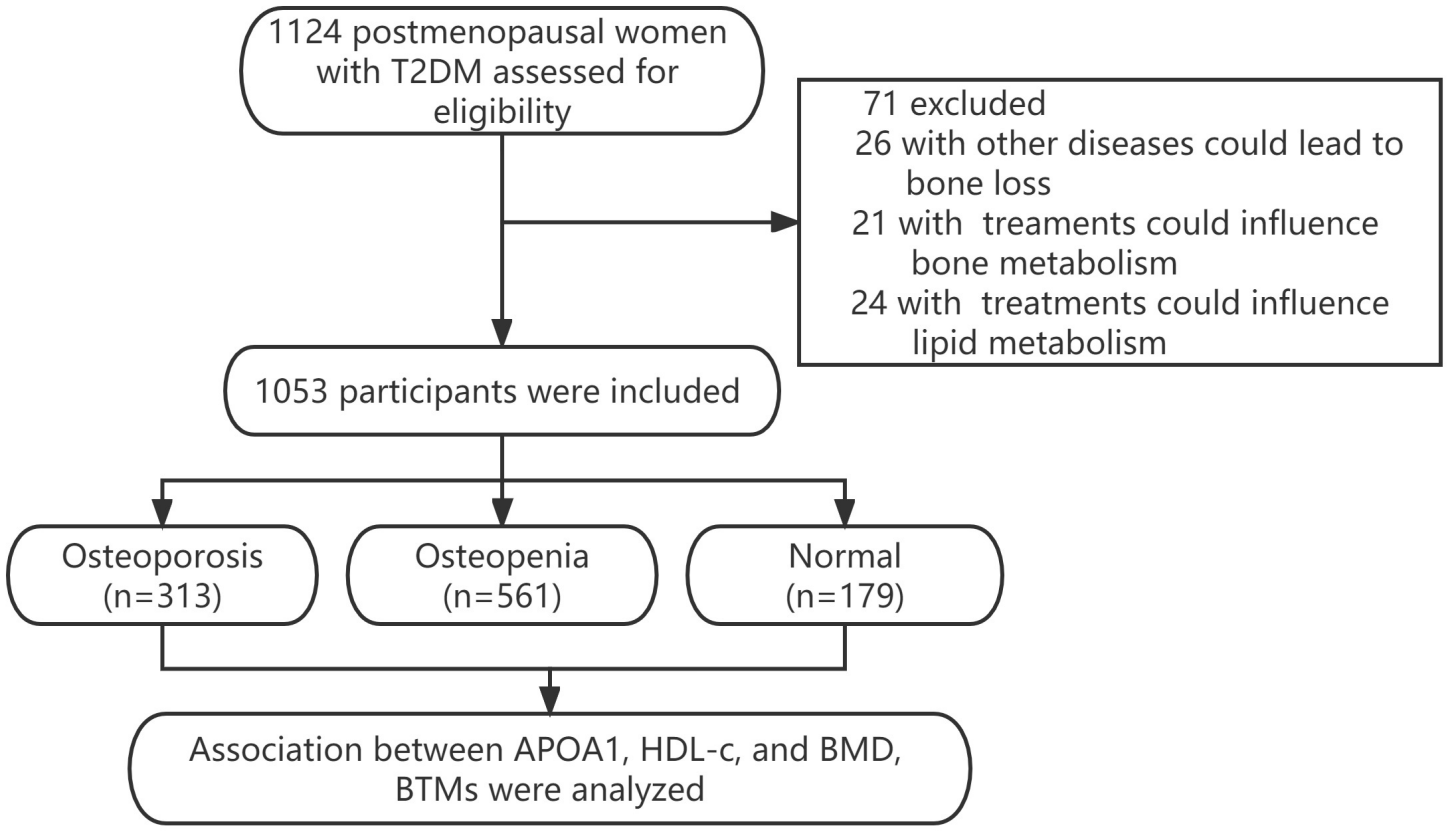
Flowchart describing the selection process of the study population in this study.

This cross-sectional study consecutively enrolled postmenopausal women with T2DM admitted to the Department of Endocrinology at the Longyan First Affiliated Hospital of Fujian Medical University between January 2020 and December 2022. Postmenopausal women were defined as participants with twelve consecutive months of amenorrhea. The diagnosis of T2DM was according to the World Health Organization criteria (2019 edition): (1) fasting blood glucose (FBG) ≥ 126 mg/dL or 2 h postprandial ≥ 200 mg/dL during oral glucose tolerance test or glycosylated hemoglobin A1c (HbA1c) ≥ 6.5% or participants with random plasma glucose ≥ 200 mg/dL accompanied with classic symptoms of hyperglycemia or hyperglycemic crisis. (2) with diabetic autoimmune antibodies negative and exclude other specific types of diabetes. Participants were excluded if they met the following criteria: (1) history of chronic or acute diseases can lead to secondary bone mass loss (i.e., chronic renal, cardiac, hepatic, thyroid, and rheumatic diseases or bone metastasis). (2) current or prior use of drugs can interfere with bone metabolism (i.e., glucocorticoids, anti-osteoporosis, anti-resorptive or hormonal replacement therapy, calcium or vitamin D supplementation, thiazolidinediones, and urate-lowering therapy). (3) with familial or congenital lipid metabolic disorder. Overall, a total of 1,124 participants were screened. 1,053 participants met the inclusion and exclusion criteria, with complete data in the final analysis. A flowchart describing the selection process of the study population in this study is presented in Figure 1. All procedures were conducted under the Declaration of Helsinki. This study was approved by the ethical committee of Longyan First Affiliated Hospital of Fujian Medical University (LY-2021-072). All participants provided informed consent.

### Clinical and biochemical parameters

The trained reviewers used a standard questionnaire to gather demographic data, and they additionally looked over previous medical records. Age, duration of diabetes, current or prior use of medicine, history of the disease, familial or congenital lipid metabolic disorder, smoking, drinking, physical activity, menopausal status, and duration of amenorrhea are all included in the demographic data. Smoking was defined as participants continually or accumulating more than 4 cigarettes a week for at least 6 months according to the guidelines for controlling and monitoring the tobacco epidemic (15). Drinking was defined as participants drinking more than once a year according to the global burden of disease study (16). Participants whose energy expenditure was less than 1.5 metabolic equivalents while awake were considered sedentary (e.g., watching television, reading, writing, or playing video games) (17). The trained research nurses performed anthropometric measurements, including blood pressure (BP), weight, and, height. Body mass index (BMI) was calculated as the weight divided by the square of height (kg/m^2^). After resting for more than 5 min, systolic blood pressure (SBP) and diastolic blood pressure (DBP) were measured on at least three occasions using an electronic sphygmomanometer with an appropriate cuff size. The final BP was determined by taking the average of three readings.

Laboratory assessments were carried out using fasting venous blood samples. After an 8-h overnight fast, blood samples were collected and placed in standardized tubes containing dipotassium ethylenedinitrilo tetra-acetic acid. An auto-biochemical analyzer (Roche Diagnostics Corporation) was used to determine biochemical parameters such as alanine aminotransferase (ALT), alkaline phosphatase (ALP), uric acid (UA), creatinine, fasting blood glucose (FBG), triglycerides (TGs), calcium, and phosphorus. Polyethylene glycol-enhanced immunoturbidimetric test (Maker, Chengdu, China) was utilized to determine ApoA1 concentrations. HbA1c was evaluated by high-performance liquid chromatography with a D10 set (Bio-RAD). Electro-chemiluminescence immunoassay (Roche Diagnostics GmbH, Germany) was used to measure bone turnover markers such, β-cross-linked C-telopeptide of type I collagen (β-CTX), osteocalcin (OC), intact parathyroid (iPTH), and 25 hydroxyvitamin D (25-OH-D). Serum thyroid stimulating hormone (TSH) levels were also measured to screen for thyroid diseases, which are frequently associated with T2DM and can interfere with bone metabolism. The homeostasis model assessment (HOMA-IR) was calculated using the formula: fasting serum insulin (U/mL) x FBG (mmol/l)/22.5.

### Assessment of bone mineral density

The dual-energy X-ray absorptiometry (Hologic, Marlborough, MA, USA) was used to assess the BMD of the participants. During the BMD measurement, participants were in the supine position. The lumbar spine (L1-L4), total hip, and femoral neck make up the BMD measurement areas. The densitometry scanning was performed by experienced radiographers who were also blinded to clinical information. *T*-scores were calculated according to the Hologic densitometry reference value. Longitudinal quality control checks were performed daily using whole-body and L1-L4 lumbar spine phantom provided by the manufacturer. Cross-calibration was performed weekly to monitor variations between the systems. The precision error was 1.0% for the BMD measurement. Postmenopausal women with a T score ≤ −2.5 or a history of bone fragility fractures, −2.5 < *T*-score ≤ −1.0, and *T*-score >-1.0 were considered to have osteoporosis, osteopenia, and normal BDM, respectively.

### Statistical analysis

The SPSS 23.0 software (SPSS Inc. IBM) was used to analyze the data. Descriptive and Discrete data are expressed as means ± standard deviation (SD) and frequency tables (N, %), respectively. Participants were divided into three groups based on the tertile of HDL-c and APOA1. A one-way analysis of variance (ANOVA) was used to compare the statistical differences among the groups. Chi-squared (χ2) test or Fisher exact test was used to compare categorical variables. The Pearson or Spearman correlation analysis was used to evaluate the main correlations between HDL-c, APOA1, and BMD, BTMs. The independent associations between HDL-c, APOA1, and BMD, BTMs was estimated by the multiple regression analysis after adjusting for potential confounding factors. The independent variables of HDL-c and APOA1 for osteoporosis was estimated by the binomial logistic regression analysis after adjusting for other confounding factors. A two-tailed value of *p* < 0.05 was considered statistically significant.

## Results

### Clinical and laboratory characteristics based on tertiles of HDL-c and APOA1

A total of 1,053 postmenopausal women with T2DM were included in this study. The mean age of participants was 56.4 ± 6.2 years, and the mean menopausal duration was 5.2 ± 2.2 years. The hypoglycemic agents type of participants used were presented in Supplementary Table 1. The clinical and laboratory characteristics of participants based on tertiles of HDL-c and APOA1 were summarized in Table 1. The ANOVA analysis showed no significant differences in age, diabetic duration, menopausal duration, HbA1c, TC, LDL-c, creatinine, ALT, and the proportion of sedentary behavior, smoking, and drinking across the HDL-c and APOA1 tertiles (*p* > 0.05). Meanwhile, groups with higher HDL-c and APOA1 have remarkably lower BMI, TG, UA, and HOMA-IR levels and a lower prevalence of hypertension (*p* < 0.05).

**Table 1:** Clinical and laboratory characteristics of participants based on tertiles of HDL-c and APOA1.

Variable	Tertiles of HDL-c			*p*	Tertiles of APOA1			*p*
	T1	T2	T3		T1	T2	T3	
Age (year)	56.7 ± 6.3	55.8 ± 6.6	56.7 ± 5.7	0.102	56.6 ± 6.7	56.1 ± 6.5	56.7 ± 5.2	0.348
Duration (year)	8.1 ± 2.5	8.2 ± 2.2	8.1 ± 2.7	0.603	8.2 ± 2.7	8.1 ± 2.1	8.1 ± 2.2	0.716
BMI (kg/m^2^)	25.9 ± 2.8^a^^b^	24.2 ± 2.3^a^^c^	22.4 ± 2.5^b^^c^	<0.001	25.6 ± 2.9^a^^b^	24.4 ± 2.5^a^^c^	22.1 ± 3.3^b^^c^	<0.001
HbA1c (%)	9.1 ± 4.5	8.9 ± 1.4	8.9 ± 1.4	0.520	9.0 ± 1.5	9.1 ± 1.2	8.9 ± 1.3	0.532
TG (mmol/L)	3.4 ± 2.3^a^^b^	2.0 ± 0.9^a^^c^	1.2 ± 0.7^b^^c^	<0.001	2.7 ± 1.6^a^^b^	2.2 ± 1.3^a^^c^	1.8 ± 1.2^b^^c^	<0.001
TC (mmol/L)	5.1 ± 1.2	5.0 ± 1.2	5.1 ± 1.1	0.408	5.2 ± 1.2^b^	5.1 ± 1.1	4.9 ± 1.1^b^	0.088
HDL-c (mmol/L)	0.84 ± 0.09^a^^b^	1.02 ± 0.07^a^^c^	1.35 ± 0.26^b^^c^	<0.001	0.96 ± 0.22^a^^b^	1.08 ± 0.21^a^^c^	1.18 ± 0.25^b^^c^	<0.001
LDL-c (mmol/L)	3.5 ± 1.0	3.6 ± 0.9	3.5 ± 1.0	0.428	3.6 ± 1.0	3.6 ± 1.0	3.5 ± 0.9	0.326
APOA1 (g/L)	0.90 ± 0.19^a^^b^	0.97 ± 0.20^a^^c^	1.05 ± 0.26^b^^c^	<0.001	0.76 ± 0.07^a^^b^	0.95 ± 0.05^a^^c^	1.21 ± 0.20^b^^c^	<0.001
UA (umol/L)	390 ± 94^a^^b^	365 ± 72^a^^c^	300 ± 70^b^^c^	<0.001	368 ± 78^a^^b^	342 ± 83^a^^c^	323 ± 98^b^^c^	<0.001
Creatinine (umol/L)	70.0 ± 13.9	68.3 ± 12.8	69.4 ± 13.2	0.229	68.6 ± 13.4	69.6 ± 13.7	69.5 ± 12.7	0.534
ALT (IU/L)	33.2 ± 9.2	33.7 ± 9.3	32.9 ± 10.6	0.554	33.9 ± 10.4	33.1 ± 9.6	32.9 ± 9.1	0.346
HOMA-IR	4.6 ± 2.1^a^^b^	4.0 ± 1.8^a^^c^	2.6 ± 1.9^b^^c^	<0.001	4.2 ± 2.2^a^^b^	3.4 ± 1.9^a^	3.6 ± 2.0^b^	<0.001
Menopausal duration (year)	5.2 ± 1.9	5.3 ± 2.1	5.1 ± 2.2	0.678	5.2 ± 2.1	5.1 ± 2.4	5.3 ± 1.7	0.716
Hypertension, *n* (%)	190 (55.9)^a^^b^	151 (43.5)^a^^c^	81 (22.4)^b^^c^	<0.001	188 (53.0)^a^^b^	144 (41.0)^a^^c^	94 (27.1)^b^^c^	<0.001
Smoking, *n* (%)	10 (2.9)	9 (2.6)	6 (1.7)	0.515	8 (2.3)	12 (3.4)	5 (1.4)	0.226
Drinking, *n* (%)	56 (16.5)	55 (15.7)	57 (15.7)	0.951	57 (16.1)	61 (17.4)	50 (14.4)	0.562
Sedentary behavior, *n* (%)	100 (29.4)	97 (27.6)	92 (25.4)	0.493	97 (27.3)	95 (27.1)	97 (28.0)	0.964

BMI, body mass index; HbA1c, Glycated hemoglobin; UA, uric acid; TG, triglyceride; TC, total cholesterol; HDL-c, high-density lipoprotein cholesterol; LDL-c, Low density lipoprotein cholesterol; APOA1, Apolipoprotein A1; HOMR-IR, Homeostasis model assessment insulin resistance.

a *p* < 0.05: T1 vs T2.

b *p* < 0.05: T1 vs T3.

c *p* < 0.05: T2 vs T3.

### BTMs and BMD of participants based on tertiles of HDL-c and APOA1

**Figure 2:**
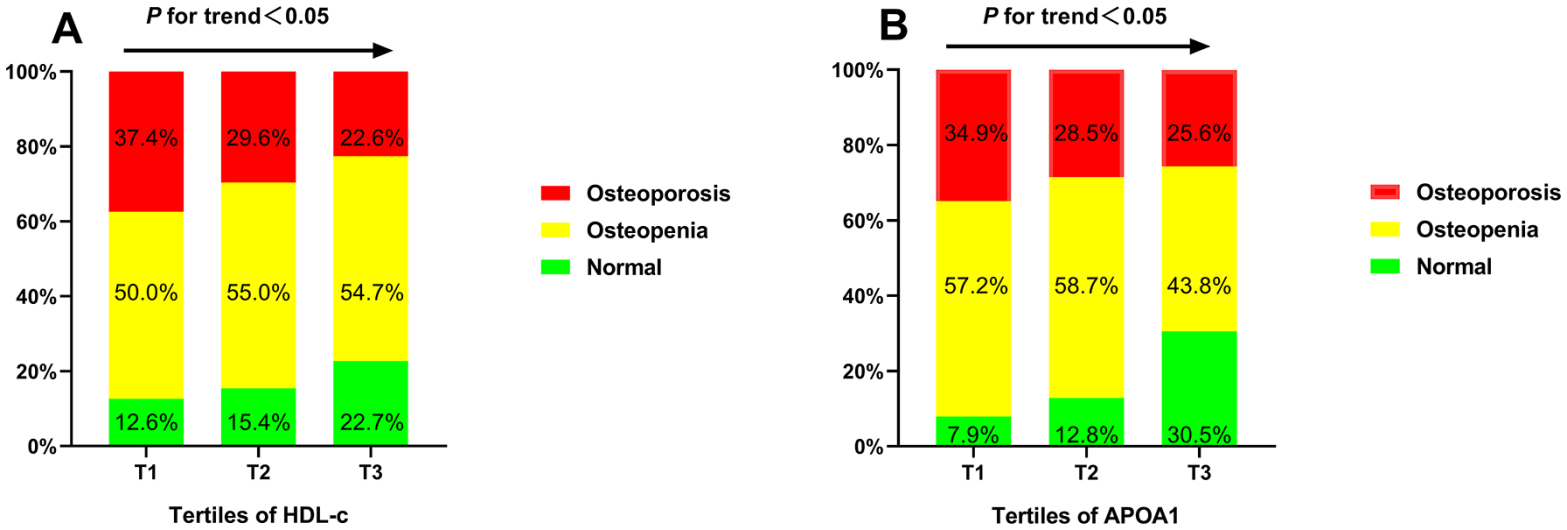
The prevalence of osteoporosis, osteopenia, and normal BMD across the HDL-c **(A)** and APOA1 **(B)** tertiles.

The prevalence of osteoporosis was 29.7% in postmenopausal with T2DM. The mean BMD was 0.89 ± 0.11 g/cm3 in L1-L4, 0.81 ± 0.09 g/cm3 in the hip, and 0.70 ± 0.12 g/cm3 in the femoral neck. The BTMs and BMD of participants based on tertiles of HDL-c and APOA1 were summarized in Table 2. The results showed no significant differences in β-CTX, 25-OH-D, iPTH, ALP, calcium and phosphorous levels, hip BMD and *T*-score, femoral neck BMD, and *T*-score across the HDL-c and APOA1 tertiles (*p* > 0.05). In addition, there were also no significant differences in levels of OC, L1-L4 BMD, and *T*-score across the HDL-c tertiles (*p* > 0.05). In contrast, groups with higher APOA1 have a remarkably more elevated level of OC, L1-L4 BMD, and *T*-score across the APOA1 tertiles (*p* < 0.05). The prevalence of osteoporosis among different tertiles of HDL-c and APOA1 is illustrated in Figure 2. The results displayed a decreasing trend in the prevalence of osteoporosis across the HDL-c and APOA1 tertiles (*p* < 0.05).

**Table 2:** BTMs and BMD of participants based on tertiles of HDL-c and APOA1.

Variable	Tertiles of HDL-c			*p*	Tertiles of APOA1			*p*
	T1	T2	T3		T1	T2	T3	
OC (ng/mL)	16.6 ± 3.7	16.9 ± 3.3	16.4 ± 3.0	0.209	15.7 ± 3.4^a^^b^	16.6 ± 3.1^a^^c^	17.6 ± 3.3^b^^c^	<0.001
β-CTX (ng/mL)	0.49 ± 0.18	0.47 ± 0.17	0.47 ± 0.24	0.491	0.48 ± 0.21	0.48 ± 0.21	0.47 ± 0.17	0.879
25-OH-D (nmol/L)	49.4 ± 22.5	48.1 ± 20.5	50.9 ± 24.5	0.240	50.3 ± 25.3	48.9 ± 21.9	49.2 ± 20.4	0.664
iPTH (ng/L)	36.0 ± 13.3^a^	33.8 ± 13.2^a^	34.8 ± 14.4	0.101	35.3 ± 15.1	35.0 ± 14.3	34.3 ± 11.1	0.538
ALP (IU/L)	77.3 ± 20.3	79.7 ± 20.3	78.6 ± 19.6	0.184	79.4 ± 21.1	78.9 ± 20.6	77.1 ± 19.1	0.289
Calcium (mmol/L)	2.31 ± 0.11	2.31 ± 0.11	2.32 ± 0.11	0.366	2.31 ± 0.10	2.32 ± 0.10	2.32 ± 0.11	0.562
Phosphorous (mmol/L)	1.22 ± 0.17	1.21 ± 0.15	1.22 ± 0.19	0.272	1.23 ± 0.17	1.21 ± 0.18	1.23 ± 0.17	0.169
L1-L4 BMD (g/cm^3^)	0.89 ± 0.11	0.89 ± 0.10	0.89 ± 0.12	0.703	0.86 ± 0.10^a^^b^	0.89 ± 0.09^a^^c^	0.92 ± 0.13^b^^c^	<0.001
L1-L4 *T*-score	−1.8 ± 1.0^b^	−1.7 ± 0.8	−1.6 ± 0.9^b^	0.028	−1.9 ± 0.8^a^^b^	−1.7 ± 0.8^a^^c^	−1.4 ± 1.2^b^^c^	<0.001
Hip BMD (g/cm^3^)	0.81 ± 0.06	0.82 ± 0.11	0.81 ± 0.10	0.642	0.82 ± 0.09	0.81 ± 0.11	0.81 ± 0.12	0.576
Hip *T*-score	−1.4 ± 0.6	−1.3 ± 0.7	−1.4 ± 0.8	0.617	−1.3 ± 0.9	−1.4 ± 0.5	−1.4 ± 0.7	0.594
Femoral neck BMD (g/cm^3^)	0.70 ± 0.13	0.71 ± 0.08	0.71 ± 0.12	0.492	0.71 ± 0.13	0.71 ± 0.10	0.70 ± 0.11	0.376
Femoral neck *T*-score	−1.3 ± 0.9	−1.2 ± 0.8	−1.2 ± 0.7	0.526	−1.2 ± 0.7	−1.2 ± 0.6	−1.3 ± 0.9	0.488

OC: osteocalcin. β-CTX: β-cross-linked C-telopeptide of type I collagen (β-CTX). 25-OH-D: 25 hydroxyvitamin D. iPTH: intact parathyroid hormone. ALP: alkaline phosphatase. BMD: bone mineral density. BTMs: bone turnover markers.

a *p* < 0.05: T1 vs T2.

b *p* < 0.05: T1 vs T3.

c *p* < 0.05: T2 vsT3.

### Main correlations of HDL-c and APOA1 with BTMs and BMD

The main correlations of HDL-c and APOA1 with BTMs and BMD are presented in Table 3. The Pearson or Spearman correlation analysis revealed that HDL-c and APOA1 were not significantly correlated with β-CTX, 25-OH-D, iPTH, ALP, calcium and phosphorous, hip BMD and *T*-score, femoral neck BMD and *T*-score (*p* > 0.05). In addition, HDL-c was also not significantly correlated with OC, L1-L4 BMD, and *T*-score (*p* > 0.05). Nevertheless, APOA1 presented a positive correlation with OC (*r* = 0.194, *p* < 0.001), L1-L4 BMD (*r* = 0.165, *p* < 0.001), and *T*-score (*r* = 0.153, *p* < 0.001).

**Table 3:** Main correlations of HDL-c and APOA1 with BTMs and BMD.

Variable	HDL-c		APOA1	
	*R*	*p*	*R*	*p*
OC (ng/mL)	−0.002	0.948	0.194	<0.001
β-CTX (ng/mL)	0.004	0.884	−0.027	0.390
25-OH-D (nmol/L)	0.033	0.287	−0.009	0.758
iPTH (ng/L)	−0.046	0.132	−0.032	0.304
ALP (IU/L)	−0.056	0.070	−0.049	0.114
Calcium (mmol/L)	0.009	0.758	0.012	0.692
Phosphorous (mmol/L)	0.019	0.537	0.038	0.217
L1-L4 BMD (g/cm^3^)	−0.018	0.555	0.165	<0.001
L1-L4 *T*-score	0.044	0.154	0.153	<0.001
Hip BMD (g/cm^3^)	0.021	0.488	−0.005	0.879
Hip *T*-score	0.010	0.746	−0.009	0.758
Femoral neck BMD (g/cm^3^)	0.017	0.583	−0.020	0.528
Femoral neck *T*-score	0.006	0.839	−0.021	0.498

OC: osteocalcin. β-CTX: β-cross-linked C-telopeptide of type I collagen (β-CTX). 25-OH-D: 25 hydroxyvitamin D. iPTH: intact parathyroid hormone. ALP: alkaline phosphatase. BMD: bone mineral density. BTMs: bone turnover markers.

### Impact of HDL-c and APOA1 on OC, L1-L4 BMD, and *T*-score

The multiple linear regression analysis was also conducted to further evaluate the associations between HDL-c and APOA1 on OC, L1-L4 BMD, and *T*-score. As shown in Table 4, after adjustment for age, BMI, diabetic duration, menopausal duration, hypertension, sedentary behavior, smoking, drinking (Model 1), HbA1c, TG, HDL-c, APOA1, LDL-c, creatinine, ALT, UA, and HOMA-IR (Model 2), APOA1 was shown to be positively correlated with OC, L1-L4 BMD, and *T*-score. APOA1 remained independently associated with OC (*β* = 0.126, *p <* 0.001), L1-L4 BMD (*β* = 0.181, *p <* 0.001), and *T*-score (*β* = 0.180, *p <* 0.001) after additional adjustment for BTMs like β-CTX, 25-OH-D, ALP, iPTH, calcium, phosphorous (Model 3), and OC (Model 4). Furthermore, there was no significant association of HDL-c with L1-L4 BMD, L1-L4 *T*-score, and OC after adjustment for Model 1, Model 2, and Model 4.

**Table 4:** Multivariate linear regression analysis between HDL-c, APOA1 and OC, L1-L4 BMD, LI-L4  *T*-score.

Variable	HDL-c		APOA1	
	*β*	*p*	*β*	*p*
OC (ng/mL)				
Model 1	−0.059	0.102	0.186	<0.001
Model 2	−0.058	0.155	0.123	<0.001
Model 3	−0.047	0.243	0.126	<0.001
L1-L4 BMD (g/cm^3^)				
Model 1	0.015	0.690	0.171	<0.001
Model 2	0.066	0.120	0.195	<0.001
Model 4	0.078	0.078	0.181	<0.001
L1-L4 *T*-score				
Model 1	0.050	0.169	0.180	<0.001
Model 2	0.084	0.053	0.202	<0.001
Model 4	0.082	0.068	0.180	<0.001

Model 1: Adjusted for age, BMI, diabetic duration, menopausal duration, hypertension, sedentary behavior, smoking and drinking. Model 2: Additional adjustment for HbA1c, TG, HDL-c, APOA1, LDL-c, creatinine, ALT,UA and HOMA-IR based on Model 1. Model 3: Additional adjustment for β-CTX, 25-OH-D, iPTH, ALP, calcium, and phosphorous based on Model 2. Model 4: Additional adjustment for OC based on Model 3.

### Impact of HDL-c and APOA1 on osteoporosis

**Figure 3:**
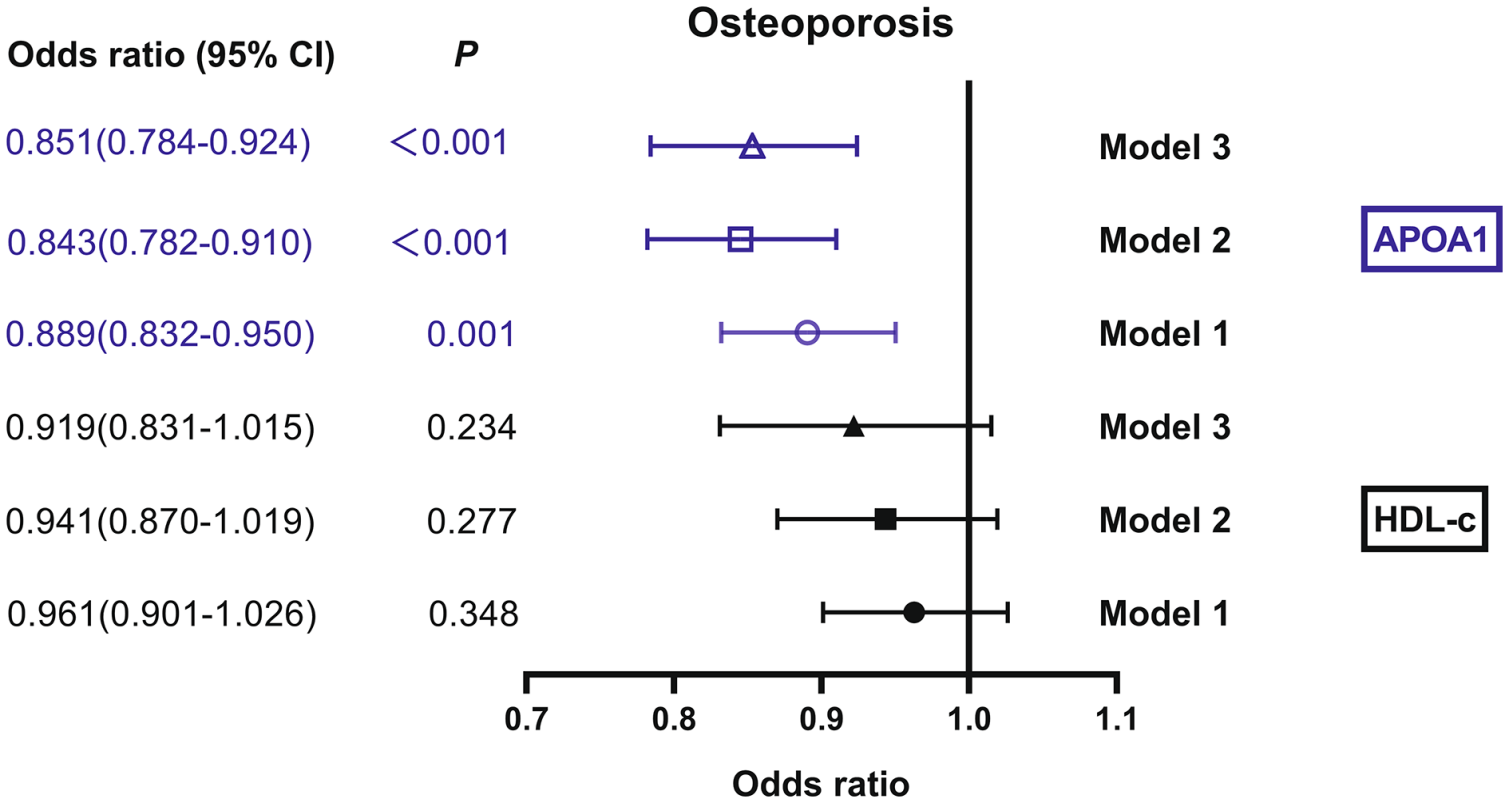
Impact of HDL-c and APOA1 on osteoporosis by binomial logistic regression analysis. Model 1: adjusted for age, BMI, diabetic duration, menopausal duration, hypertension, sedentary behavior, smoking, and drinking. Model 2: additional adjustment for HbA1c, TG, HDL-c, APOA1, LDL-c, creatinine, ALT, UA, and HOMA-IR. Model 3: further adjustment for OC, β-CTX, 25-OH-D, iPTH, ALP, calcium, and phosphorous.

The binomial logistic regression analysis was also conducted to assess the independent variables of HDL-c and APOA1 for osteoporosis. As shown in Figure 3, the results showed that the risk of osteoporosis significantly decreased with the increase of APOA1. After adjustment for age, BMI, diabetic duration, menopausal duration, hypertension, sedentary behavior, smoking, and drinking (model 1), APOA1 was independently correlated with osteoporosis, and the ORs (95%CI) was 0.889 (0.832–0.950). A significant association between osteoporosis and APOA1 was also observed after additional adjustment for HbA1c, TG, HDL-c, APOA1, LDL-c, creatinine, ALT, UA, and HOMA-IR (model 2), and the ORs (95%CI) was 0.843 (0.782–0.910) respectively. Furthermore, the ORs remained significant after further adjustment for bone turnover markers like OC, β-CTX, 25-OH-D, iPTH, ALP, calcium, and phosphorous (model 3), and the ORs (95%CI) was 0.851 (0.784–0.924). In contrast, there was no significant association between HDL-c and osteoporosis in any models.

### Values of HDL-c and APOA1 in identifying osteoporosis

**Figure 4:**
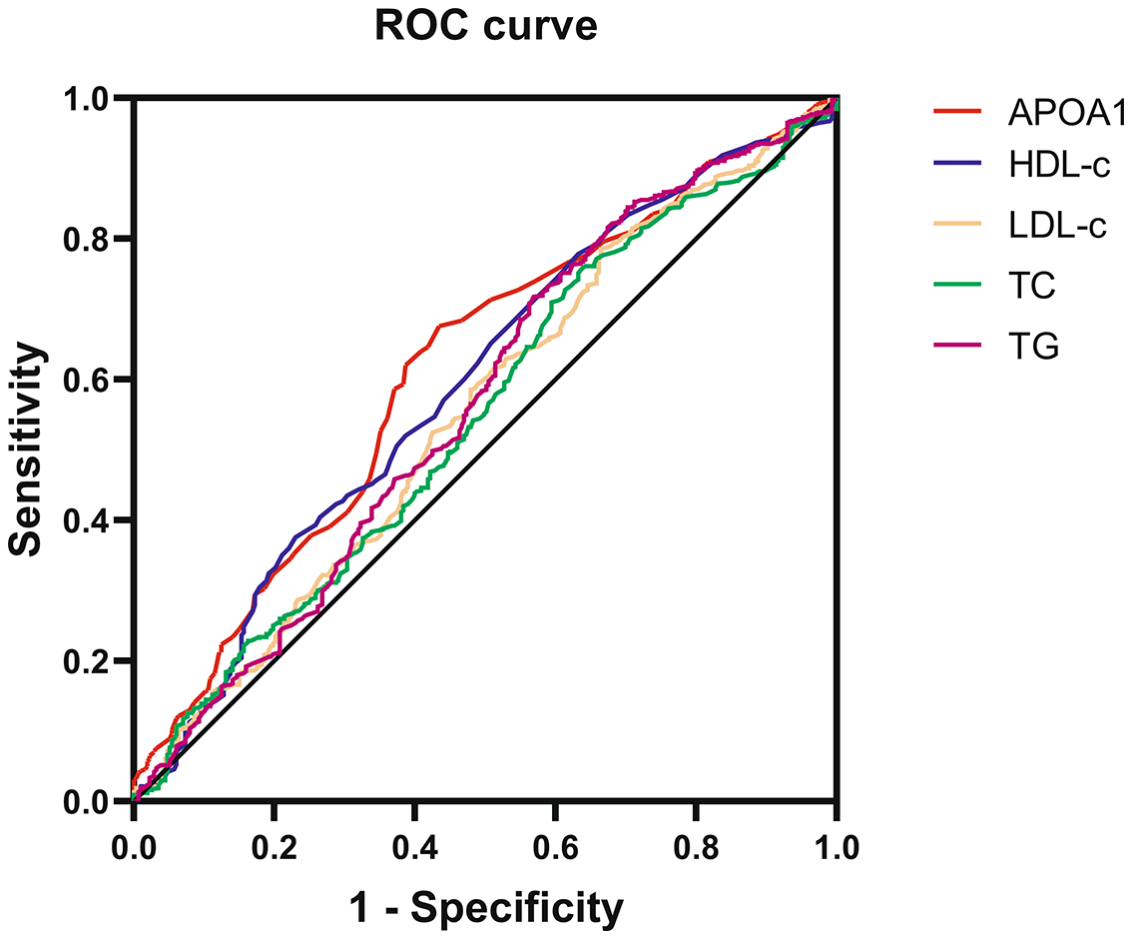
Receiver operating characteristic curves for the cutoff value of serum lipids identifying osteoporosis.

Figure 4 shows the performance for evaluating the value of different serum lipids for osteoporosis risk. The results showed that the AUC of APOA1 was larger than other serum lipids (*p* < 0.05). The AUC (95% CI) of APOA1 in identifying osteoporosis was 0.615 (0.577–0.652). The optimal cut-off value of APOA1 was 0.89 g/L, with a sensitivity of 56.5% and specificity of 67.9% (Table 5).

**Table 5:** ROC curve analysis of serum lipids in identifying osteoporosis.

Variables	AUC(95% CI)	Cut-Off value	Sensitivity (%)	Specificity (%)
APOA1	0.615 (0.577–0.652)	0.89	56.5	67.6
HDL-c	0.584 (0.546–0.622)	1.15	77.0	37.6
LDL-c	0.555 (0.516–0.593)	2.86	33.5	78.5
TC	0.548 (0.510–0.587)	4.32	36.1	75.9
TG	0.567 (0.527–0.605)	2.37	56.9	28.2

TG, triglyceride; TC, total cholesterol; HDL-c, high-density lipoprotein cholesterol; LDL-c, low density lipoprotein cholesterol; APOA1, Apolipoprotein A1.

## Discussion

Emerging evidence demonstrated that disturbances in lipid metabolic pathways could affect osteoblasts differentiation, leading to bone mass loss. Nevertheless, the association between HDL-c, APOA1, and BMD, BTMs in Chinese postmenopausal women with T2DM remained uncertain. This cross-sectional study revealed that APOA1 positively correlates with OC, L1-L4 BMD, and *T*-score after adjusting for potential confounding factors. In addition, APOA1 is also shown to be independently associated with lower odds of osteoporosis. In contrast, there was no significant association between HDL-c and BMD, BTMs. Furthermore, APOA1 has a more considerable osteoporosis-identifying value than other serum lipids.

Osteoporosis and T2DM are prevalent diseases that have become major public health concerns in the increasingly aging population. Epidemiological studies reported that the prevalence of osteoporosis is approximately 30 to 40% in Chinese postmenopausal with T2DM (18). The prevalence of osteoporosis is 29.4% in our study, which is consistent with the previous studies. Lipid metabolites are widely distributed throughout the human body and are essential in several metabolic pathways. T2DM is a kind of metabolic disorder that is accompanied by dyslipidemia. The main manifestations of lipid changes in T2DM are reduced HDL-c and elevated LDL-c. Besides the well-recognized anti-atherogenic effects, recent advances in bone metabolism and lipid highlighted that HDL-c plays a functional role in bone metabolism. The changes in HDL-c can lead to significant disruptions in the bone microenvironment. Reduced HDL levels have been associated with developing an inflammatory micro-environment (19). It is well-recognized that chronic inflammation strongly affects bone remodeling, affects the function of osteoblasts and osteoclast functions to varying degrees, and therefore plays a central role in developing bone-related metabolic pathology (20, 21). Triantaphyllidou et al. found a novel function of HDL in the pathogenesis of degenerative and metabolic bone diseases. Perturbations in the HDL metabolic pathway predispose to bone mass loss by inhibiting osteoblasts differentiation and modification of specific bone-associated chemokines and signaling cascades (12). Elevated bone marrow adiposity can affect osteocytes to varying degrees and is involved in the pathogenesis of bone-related pathologies, such as osteoporosis. Novel data from experimental mice or cells suggest that dysfunctional HDL status can influence the bone marrow microenvironment and the osteoblastic niche (22, 23). These findings may indicate that low and dysfunctional HDL may lead to reduced bone mass and impaired bone quality, resulting in an increased prevalence of osteoporosis.

Despite most studies confirming that dysfunctional HDL-c can directly affect bone metabolic homeostasis in mice or *in vivo*, the exact association between HDL-c level and BMD in real-world studies is inconsistent. In Asian postmenopausal women, two cross-sectional studies that enrolled more than 1,000 Korean women suggested a positive association between HDL-c and lumbar spine BMD in postmenopausal women (24, 25). In contrast, no association was found between HDL-c and BMD in another study that enrolled 355 postmenopausal Korean women (26). In western postmenopausal women, a cohort study that enrolled more than 3,000 Swedish postmenopausal women showed that HDL-c was negatively correlated with wrist BMD (27). Nevertheless, two cohort studies enrolled American and Danish postmenopausal women did not find an association between HDL-c and BMD (28, 29). There was also no significant association between HDL-c and BMD in Chinese postmenopausal women in two cohort studies that enrolled more than 2000 postmenopausal women (30, 31). Genetic background, age, eating habits, and hormonal and metabolic status may be responsible for this inconsistency. There is a lack of epidemiological data on the correlation between HDL-c, APOA1 levels, and BMD in Chinese postmenopausal women. In our study, after adjusting for several potential confounding factors, there was no significant association between HDL-c and BMD, BTMs. This finding is consistent with the above two studies involving Chinese postmenopausal women. It is worth noting that HDL-c functionality relies on the particle function of HDL apolipoprotein and lipid content rather than HDL levels.

APOA1 is the main protein component of HDL-c that plays a crucial role in the biogenesis and functions of HDL-c. Besides the well-documented cardioprotective functions, APOA1 is also identified to have additional beneficial functions like anti-inflammatory, anti-atherogenic, anti-apoptotic, and anti-thrombotic (32). Recent data from experimental mice also confirmed that ApoA1 deficiency generates changes in the bone cell precursor population, can increase adipoblast and decreases osteoblast production (13). In addition, APOA1 deficiency can also influence bone metabolism by reshaping bone marrow adipocyte phenotypic and molecular characteristics (33, 34). OC is a product of osteoblasts that accumulates in the extracellular matrix of bone. Serum OC level can reflect the osteoblast activity and the rate of bone formation. Our study showed that APOA1 is positively associated with OC after adjustment for potential confounding factors. This finding may suggest that serum APOA1 level can reflect the osteoblasts’ activity. Furthermore, APOA1 is also shown to be independently correlated with L1-L4 BMD and lower odds of osteoporosis. These findings may indicate that APOA1 has an anti-osteoporosis effect by regulating the activity of osteoblasts. Meanwhile, there remains a question of why APOA1 is only associated with L1-L4 BMD. More studies are needed to confirm these findings and illustrate the potential mechanisms.

### Strength and limitation

This study adjusted several potential confounding variables in the final analysis and included enough more than 1,000 participants can represent the population of Chinese postmenopausal women with T2DM. Several limitations are needed to mention in our study. First, this study was designed as a cross-sectional study that cannot directly reflect the associations between HDL-c, APOA1 and, BTMs, BMD. Second, the study population is Chinese postmenopausal women with T2DM, and these associations may not apply to other races, hormonal status, and metabolic status. Third, APOA1 seemed to have a more significant identifying value of osteoporosis than other serum lipids, and more studies with enough follow-up to confirm these findings.

## Conclusion

Work completed on data showed that the associations between HDL-c, APOA1 level, and BMD in Chinese postmenopausal with T2DM remained uncertain. Our study showed that serum APOA1 is positively associated with OC, L1-L4 BMD, and *T*-score rather than HDL-c. Furthermore, APOA1 is also shown to be independently associated with lower odds of osteoporosis. These findings may indicate that APOA1 has an anti-osteoporosis effect by regulating the activity of osteoblasts. At the same time, more studies are needed to confirm these findings further and illustrate the potential mechanisms.

## Data availability statement

The original contributions presented in the study are included in the article/Supplementary material, further inquiries can be directed to the corresponding authors.

## Ethics statement

The studies involving human participants were reviewed and approved by the Ethical Committee of Longyan First Affiliated Hospital of Fujian Medical University. The patients/participants provided their written informed consent to participate in this study.

## Author contributions

WW took charge of the software and contributed to writing— the original draft. WW, ZC, FL, XG, and MT conducted the investigation. XG and MT contributed to data curation and writing editing. All authors contributed to the article and approved the submitted version.

## Conflict of interest

The authors declare that the research was conducted in the absence of any commercial or financial relationships that could be construed as a potential conflict of interest.

## Publisher’s note

All claims expressed in this article are solely those of the authors and do not necessarily represent those of their affiliated organizations, or those of the publisher, the editors and the reviewers. Any product that may be evaluated in this article, or claim that may be made by its manufacturer, is not guaranteed or endorsed by the publisher.
