# Supplementary material for: Apolipoprotein A1 is associated with osteocalcin and bone mineral density rather than high-density lipoprotein cholesterol in Chinese postmenopausal women with type 2 diabetes mellitus
Source: Front Med (Lausanne). 2023 Jun 15;10:1182866. doi: 10.3389/fmed.2023.1182866 (PMC10308019; doi:10.3389/fmed.2023.1182866)
Supplement: Supplementary file 1 [file Table_1.DOCX]

| **Supplementary Table 1.** Hypoglycemic agents type of participants used . | |
| --- | --- |
| Insulin，n(%) | 168(15.9) |
| GLP-1 RAs，n(%) | 94(8.9) |
| Metformin，n(%) | 682(64.8) |
| SGLT-2，n(%) | 177(16.4) |
| DPP4i，n(%) | 235(22.3) |
| Sulfonylurea，n(%) | 346(32.9) |
| AGI，n(%) | 483(45.9) |
| TZDs，n(%) | 86(8.7) |
| Number of other OADs | |
| 0 | 36(3.4) |
| 1 | 184(17.5) |
| 2 | 488(46.3) |
| 3 | 345(32.8) |
| GLP-1 RAs: glucagon-like peptide-1 receptor agonists. SGLT-2: sodium-dependent glucose transporters 2 inhibitors. DPP4i: dipeptidyl peptidase-4 inhibitors. AGI:a-glucosidase inhibitors. TZDs: thiazolidinediones. OADs: oral hypoglycemic agents. | |
